# Rheumatoid arthritis and the risk of chronic kidney diseases: a Mendelian randomization study

**DOI:** 10.3389/fmed.2024.1360026

**Published:** 2024-05-16

**Authors:** Zhaoyu Jiang, Lin Chen, Aihui Liu, Jiaping Qi, Jing Wang, Yixuan Li, Huan Jiang, Ju Zhang, Shan Huang, Chengliang Mao, Zhenhua Ying

**Affiliations:** ^1^Zhejiang Province People’s Hospital, Affiliated People's Hospital, Hangzhou Medical College, Hangzhou, China; ^2^Department of Rheumatology and Immunology of Zhejiang Provincial People's Hospital, Center for General Practice Medicine, Hangzhou, China; ^3^Zhejiang Provincial Key Laboratory of Traditional Chinese Medicine for Arthritis Diagnosis and Treatment, Hangzhou, China

**Keywords:** rheumatoid arthritis, chronic kidney diseases, Mendelian randomization analysis, genome-wide association studies, inverse-variance weighted

## Abstract

**Background:**

The extra-articular lesions of rheumatoid arthritis (RA) are reported to involve multiple organs and systems throughout the body, including the heart, kidneys, liver, and lungs. This study assessed the potential causal relationship between RA and the risk of chronic kidney diseases (CKDs) using the Mendelian randomization (MR) analysis.

**Method:**

Independent genetic instruments related to RA and CKD or CKD subtypes at the genome-wide significant level were chosen from the publicly shared summary-level data of genome-wide association studies (GWAS). Then, we obtained some single-nucleotide polymorphisms (SNPs) as instrumental variables (IVs), which are associated with RA in individuals of European origin, and had genome-wide statistical significance (p5 × 10^−8^). The inverse-variance weighted (IVW) method was the main analysis method in MR analysis. The other methods, such as weighted median, MR–Egger, simple mode, and weighted mode were used as supplementary sensitivity analyses. Furthermore, the levels of pleiotropy and heterogeneity were assessed using Cochran’s Q test and leave-one-out analysis. Furthermore, the relevant datasets were obtained from the Open GWAS database.

**Results:**

Using the IVW method, the main method in MR analysis, the results showed that genetically determined RA was associated with higher risks of CKD [odds ratio (OR): 1.22, 95% confidence interval (CI) 1.13–1.31; *p* < 0.001], glomerulonephritis (OR: 1.23, 95% CI 1.15–1.31; *p* < 0.000), amyloidosis (OR = 1.43, 95% CI 1.10–1.88, *p* < 0.001), and renal failure (OR = 1.18, 95% CI 1.00–1.38, *p* < 0.001). Then, using multiple MR methods, it was confirmed that the associations persisted in sensitivity analyses, and no pleiotropy was detected.

**Conclusion:**

The findings revealed a causal relationship between RA and CKD, including glomerulonephritis, amyloidosis, and renal failure. Therefore, RA patients should pay more attention to monitoring their kidney function, thus providing the opportunity for earlier intervention and lower the risk of progression to CKDs.

## Introduction

Rheumatoid arthritis (RA) is a common autoimmune disease associated with synovial tissue proliferation and inflammation of the joint tissue. It can also cause damage to other tissues and organs such as the heart, kidneys, lungs, and nervous system, leading to serious systemic disorders (1, 2). Approximately 25% of RA patients develop chronic kidney disease (CKD) (3, 4), which is proportionally higher than that of healthy individuals (5).

CKD is a common chronic disease characterized by long-term loss of kidney function with an extremely high mortality rate (6). It affects millions of patients worldwide (7). The worldwide burden of CKD is increasing yearly, with nearly 10% of the adult population worldwide affected by one of its subtypes (8, 9). There are several causes of CKD; however, diabetes, glomerulonephritis, and cystic kidney disease are among the more common and well-studied causes (10). However, the definitive influential factors of CKD have still not been fully investigated.

Recently, a few articles addressed the relationship between RA and CKD. However, from a previous report, most cases can be divided into two categories: chronic inflammation (such as secondary kidney atherosclerosis and amyloidosis) and drug-induced kidney diseases (11). Furthermore, previous studies have shown that elevated levels of inflammatory markers, including C-reactive protein (CRP), in the early clinical stages are related to future CKD (12, 13). Thus, it can be deemed that there are various causes of RA with kidney disease, but the definitive mechanism still needs to be studied.

Mendelian randomization (MR) analysis is a method used in epidemiology to research the causal relationship between genetic exposure factors and outcomes (14, 15). The MR analysis employs SNPs as IVs for exposure, thus it can effectively avoid the interference factors of relevant trait changes and has high scientific value. To explore the potential causal relationship between RA and CKD, the MR analysis was used to study whether RA affects the risk of CKDs from a genetic perspective.

## Materials and methods

### Study design

**Figure 1:**
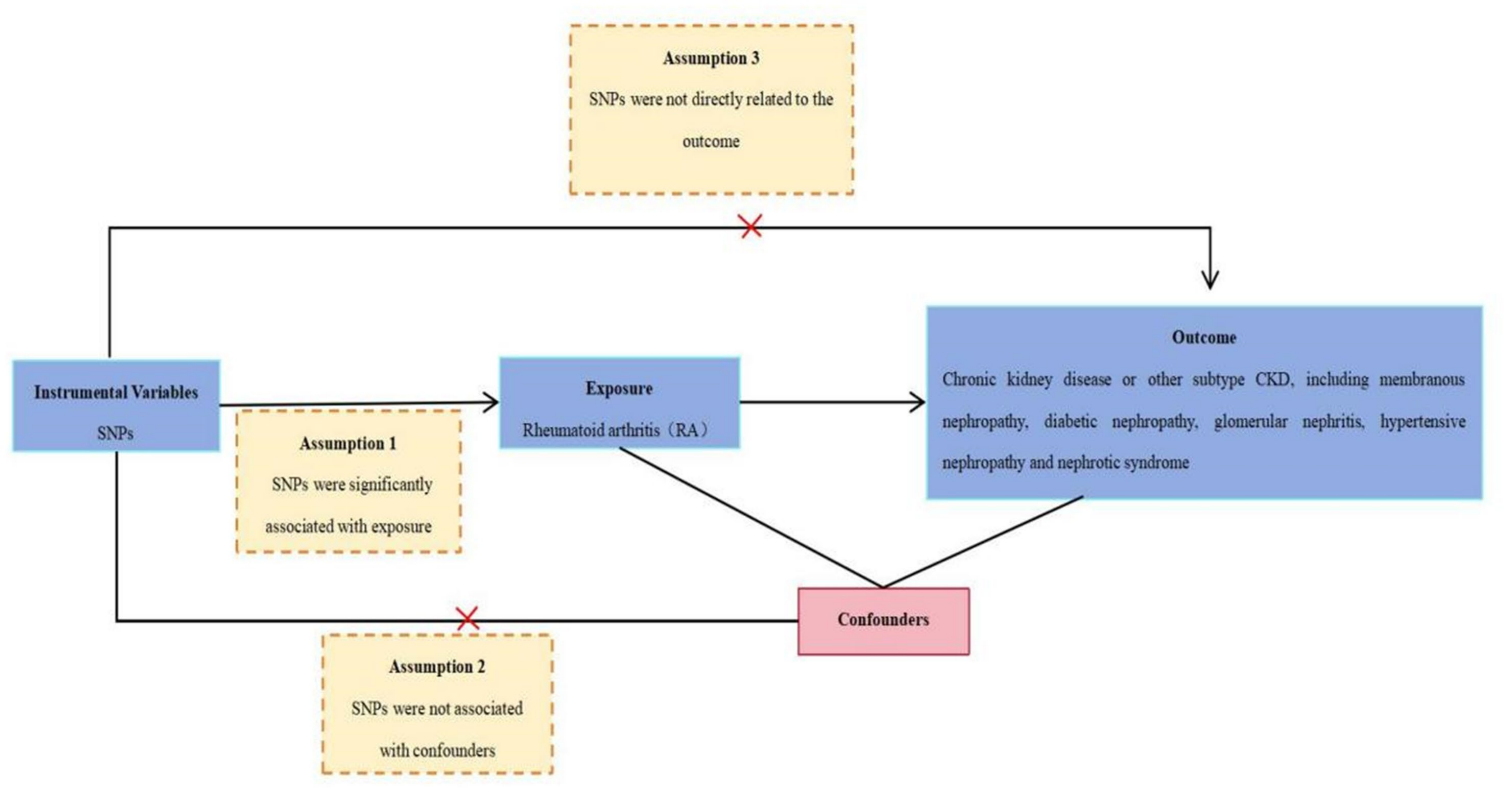
Schematic representation of an MR analysis. Three basic assumptions of MR analysis: Assumption 1: SNPs were significantly associated with RA. Assumption 2: SNPs were not associated with the confounders. Assumption 3: SNPs were not directly related to CKD. MR, Mendelian randomization; SNPs, single-nucleotide polymorphisms. IVs, instrumental variables selection. RA, rheumatoid arthritis. CKD, chronic kidney disease.

The study analyzed the causal relationship between RA and CKD using the MR analysis. RA as an exposure factor and CKD or other different subtypes of CKD (including membranous nephropathy, diabetic nephropathy, hypertensive renal disease, glomerulonephritis, nephrotic syndrome, amyloidosis, and renal failure) as outcome factors. These summary-level data, from genome-wide association studies (GWASs), can be obtained from the IEU Open database.^1^ This analysis adhered to the Strengthening the Reporting of Observational Studies in Epidemiology (STROBE)-MR Statement (16, 17). The study design overview is presented in Figure 1.

### Data source of disease

The summary-level GWAS data of RA, CKD, and other subtypes of CKD were obtained from the Open GWAS database. To minimize the bias from population stratification, we decided to incorporate the summary data from the population of European ancestry in this study. A description of the details of the data source is summarized in Table 1.

**Table 1:** Description of data used for these diseases.

Trait	GWAS ID	ncase	N controls	Number of SNPs	Population
Rheumatoid arthritis	ebi-a-GCST90018910	8,255	409,001	24,175,266	European
Chronic kidney disease	finn-b-N14_CHRONKIDNEYDIS	3,902	212,841	16,380,459	European
Membranous nephropathy	ebi-a-GCST010005	2,150	5,829	5,327,688	European
Diabetic nephropathy	ebi-a-GCST90018832	1,032	451,248	24,190,738	European
Hypertensive Renal Disease	finn-b-I9_HYPTENSRD	462	162,837	16,380,163	European
Glomerulonephritis	finn-b-GLOMER_NEPHRITIS	4,613	214,179	16,380,466	European
Nephrotic syndrome	ebi-a-GCST90018884	775	475,255	24,196,233	European
Amyloidosis	finn-b-E4_AMYLOIDOSIS	226	197,259	16,380,377	European
Renal failure	finn-b-N14_RENFAIL	5,951	212,841	16,380,466	European

GWAS, genome-wide association studies; SNPs, single-nucleotide polymorphisms.

### Instrumental variable selection

This study extracted SNPs, as IVs related to RA, with genome-wide significance levels (*p* < 5 × 10^−8^). Thereafter, the threshold was set for linkage disequilibrium at R^2^ < 0.001 to exclude the effect of linkage disequilibrium. Furthermore, GWAS Catalog^2^ was used to verify whether the included SNPs were related to confounders. SNPs that were not related to confounders were selected for further analysis. Finally, the *F* value was calculated to assess the strength of the IVs, and an F-value < 10 indicated a significant bias in causality estimates (18). Thus, an F-value > 10 was selected in this study.

### Ethical approval

The GWAS summary data were obtained from published studies in the GWAS open database approved by the institutional review boards. Therefore, this study did not need to acquire ethical approval.

### Mendelian randomization analysis

The genetic correlations of RA with CKD and CKD subtypes were based on computation using data from the IEU Open GWAS database. Based on these data, this study utilized SNPs as IVs to evaluate the etiological relationship between RA and CKD and other CKD subsets. The random-effects IVW model was the main statistical approach, complemented by other sensitivity analyses, including MR Egger, weighted median, simple mode, weighted mode, Cochran’s Q, and leave-one-out analysis.

The IVW method is a predominantly dependent method that amounts to a correlation-weighted linear regression between variables by calculating the ORs and the 95% CIs for each additional unit in logarithmic probability (19). The relationship between the outcome and exposure variables was subjected to a weighted linear regression, combining the Wald ratio estimates for each of the meta-analytic IVs in a meta-analytic manner (20). The MR–Egger method can be used to detect and correct for possible unbalanced pleiotropy, although its estimates are usually not significant (21). Finally, the study used Cochrane’s Q and leave-one-out analysis to evaluate the polysemy and heterogeneity levels. A *p*-value of >0.05 indicates no heterogeneity. The p-value of the MR-Egger intercept was used as an indicator of pleiotropy in one analysis of SNP estimation (21). A *p*-value of <0.05 suggests horizontal pleiotropy between the exposure and outcome factors, indicating that the outcome remains even in the absence of the exposure factor.

### Statistical analysis

In this study, all the statistical examinations were two-sided tests, and R Studio was used to calculate the genetic relevance. The MR analysis was conducted using the “TwoSampleMR” (22) packages in R Studio (R version 4.3.1). A *p*-value of <0.05 was considered significant.

## Results

### The association between RA and CKD

**Figure 2:**
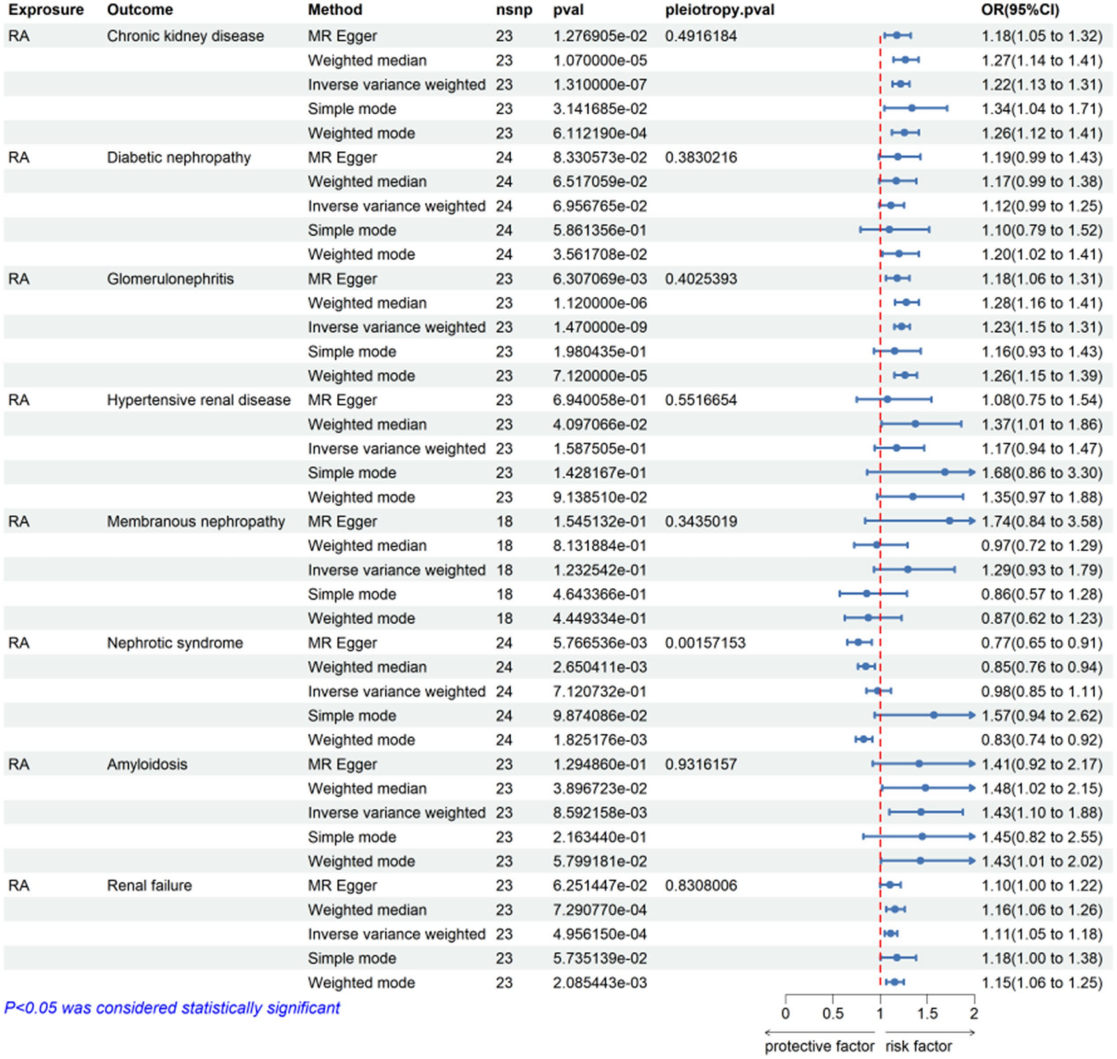
Mendelian randomization for the association of rheumatoid arthritis on CKD and other CKD subsets. RA, rheumatoid arthritis; nsnp, number of SNPs used in MR; OR, odds ratio; CI, confidence interval.

**Figure 3:**
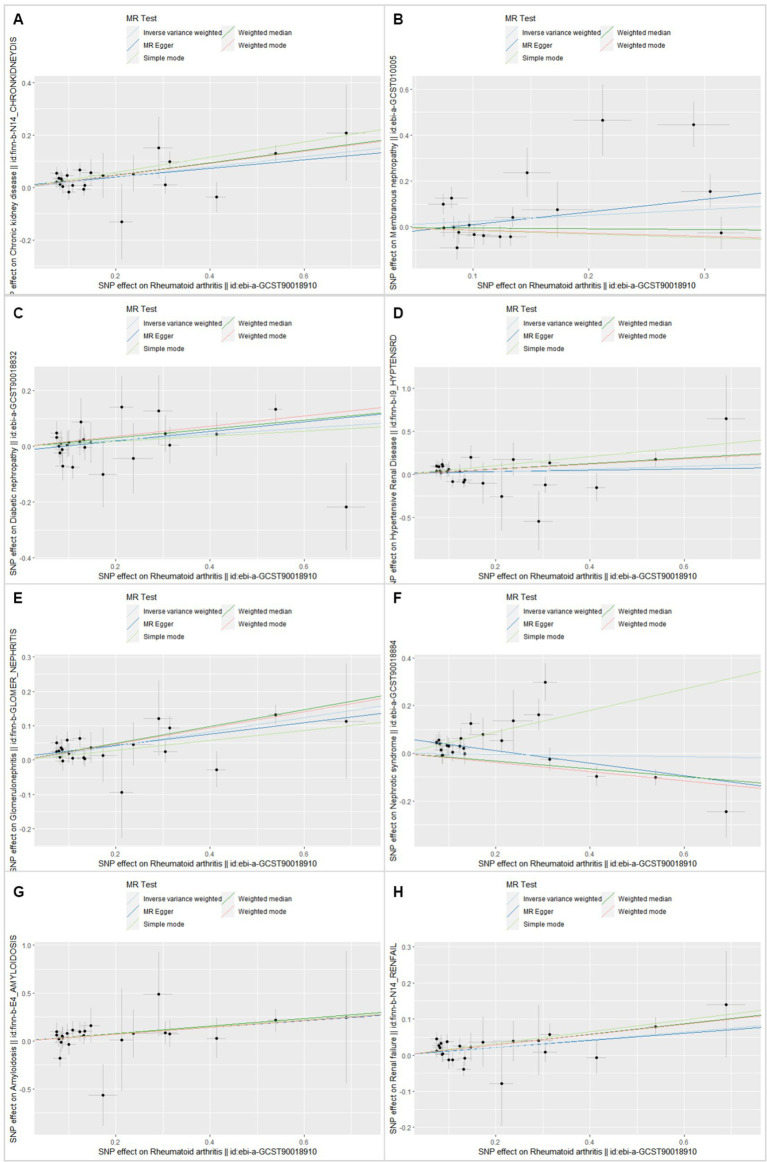
Scatter plot of single-nucleotide polymorphism (SNP) potential effects on rheumatoid arthritis vs. CKD and its subsets. The 95% CI for the effect size on CKD/CKD subsets is shown as vertical lines, and the 95% CI for the effect size on rheumatoid arthritis is shown as horizontal lines. The slope of fitted lines represents the estimated Mendelian randomization effect per method. **(A)** Chronic kidney disease. **(B)** Membranous nephropathy. **(C)** Diabetic nephropathy. **(D)** Hypertensive renal disease. **(E)** Glomerulonephritis. **(F)** Nephrotic syndrome. **(G)** Amyloidosis. **(H)** Renal failure.

**Figure 4:**
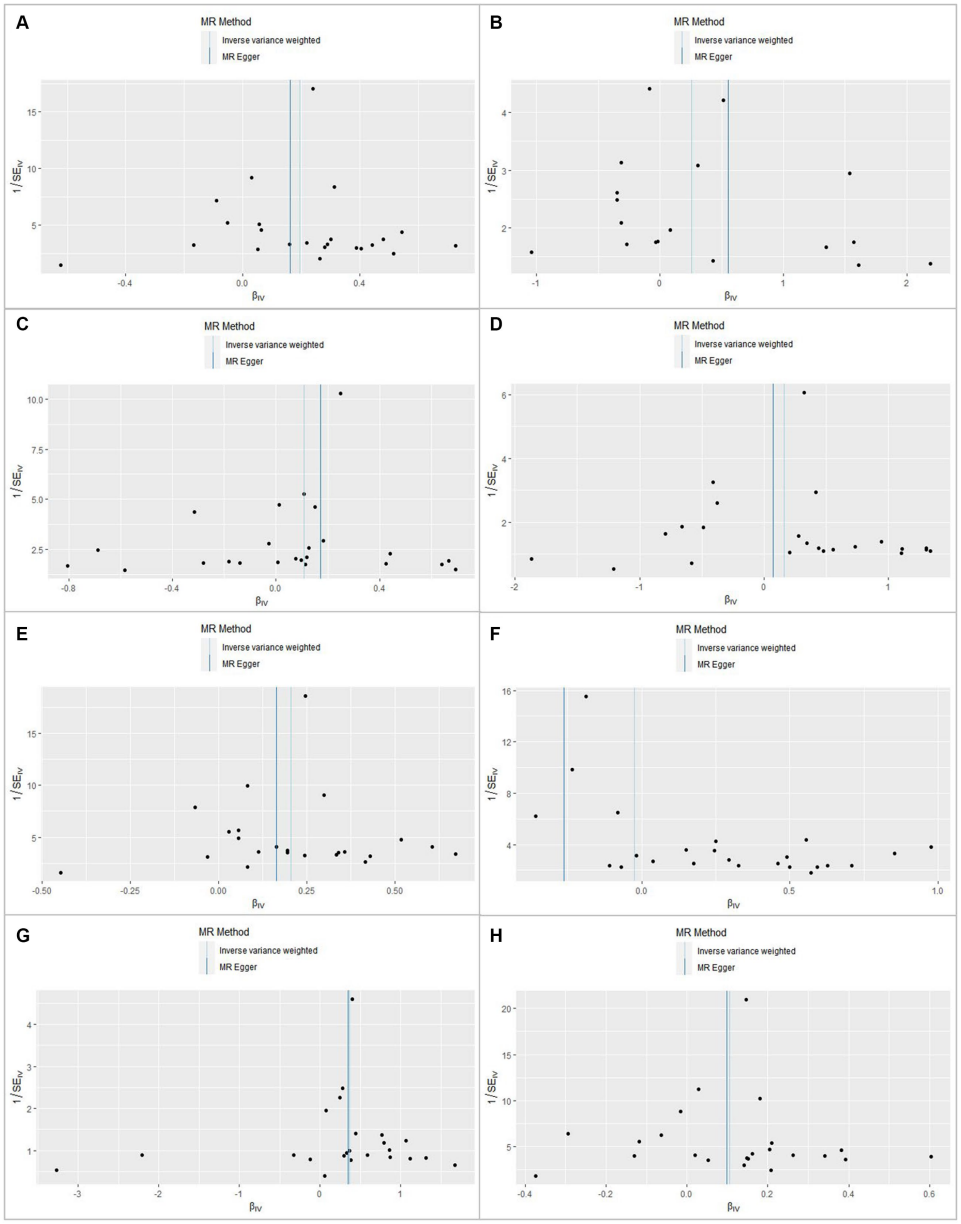
Funnel plot for rheumatoid arthritis shows the estimation using the inverse of the standard error of the causal estimate with each SNP as a tool. The vertical line represents the estimated causal effect obtained using the IVW and MR–Egger methods. **(A)** Chronic kidney disease. **(B)** Membranous nephropathy. **(C)** Diabetic nephropathy. **(D)** Hypertensive renal disease. **(E)** Glomerulonephritis. **(F)** Nephrotic syndrome. **(G)** Amyloidosis. **(H)** Renal failure.

**Figure 5:**
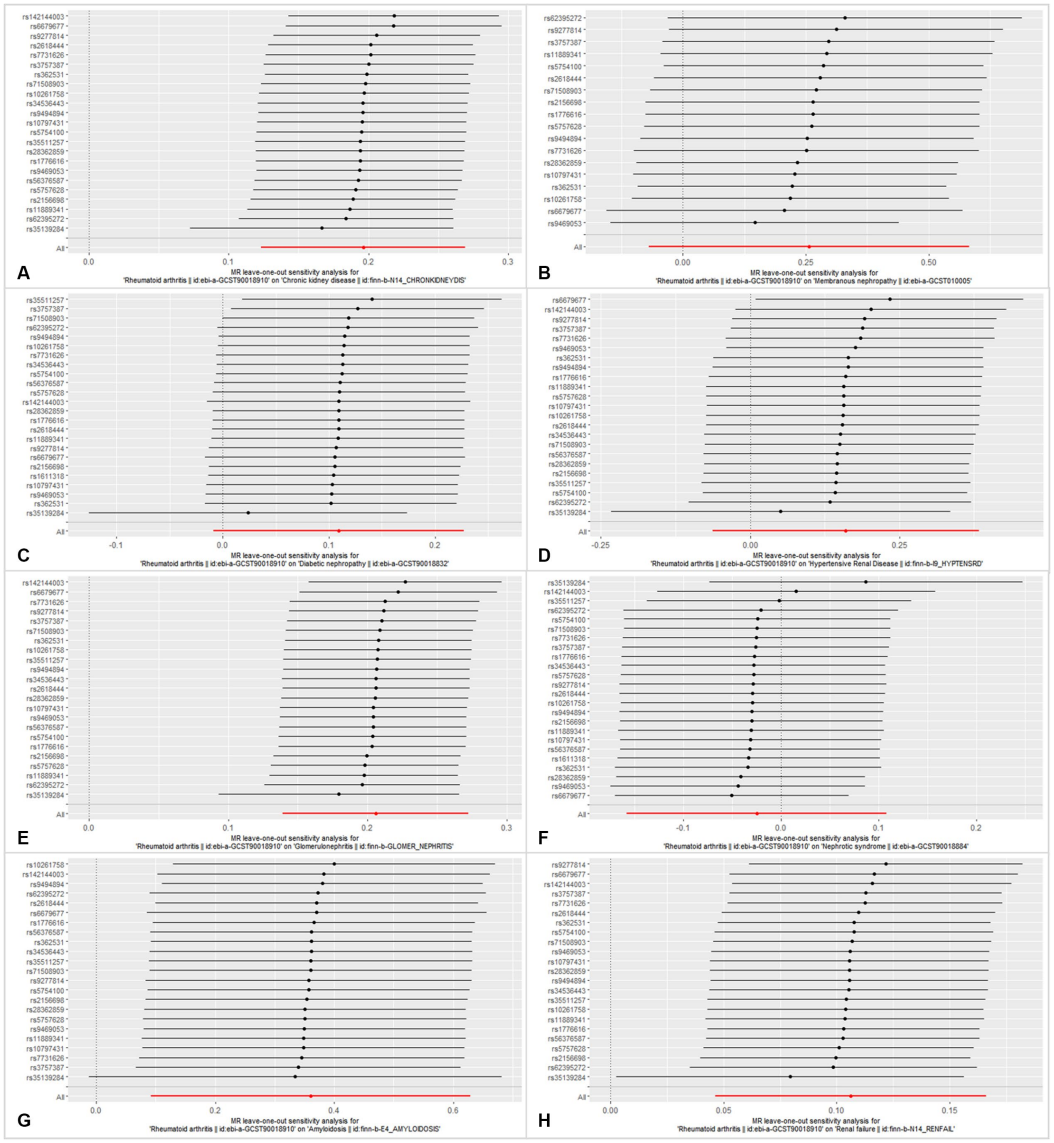
Leave-one-out analysis of each SNP associated with CKD and other CKD subtypes, and the red lines represent estimations from the IVW test. **(A)** Chronic kidney disease. **(B)** Membranous nephropathy. **(C)** Diabetic nephropathy. **(D)** Hypertensive renal disease. **(E)** Glomerulonephritis. **(F)** Nephrotic syndrome. **(G)** Amyloidosis. **(H)** Renal failure.

**Figure 6:**
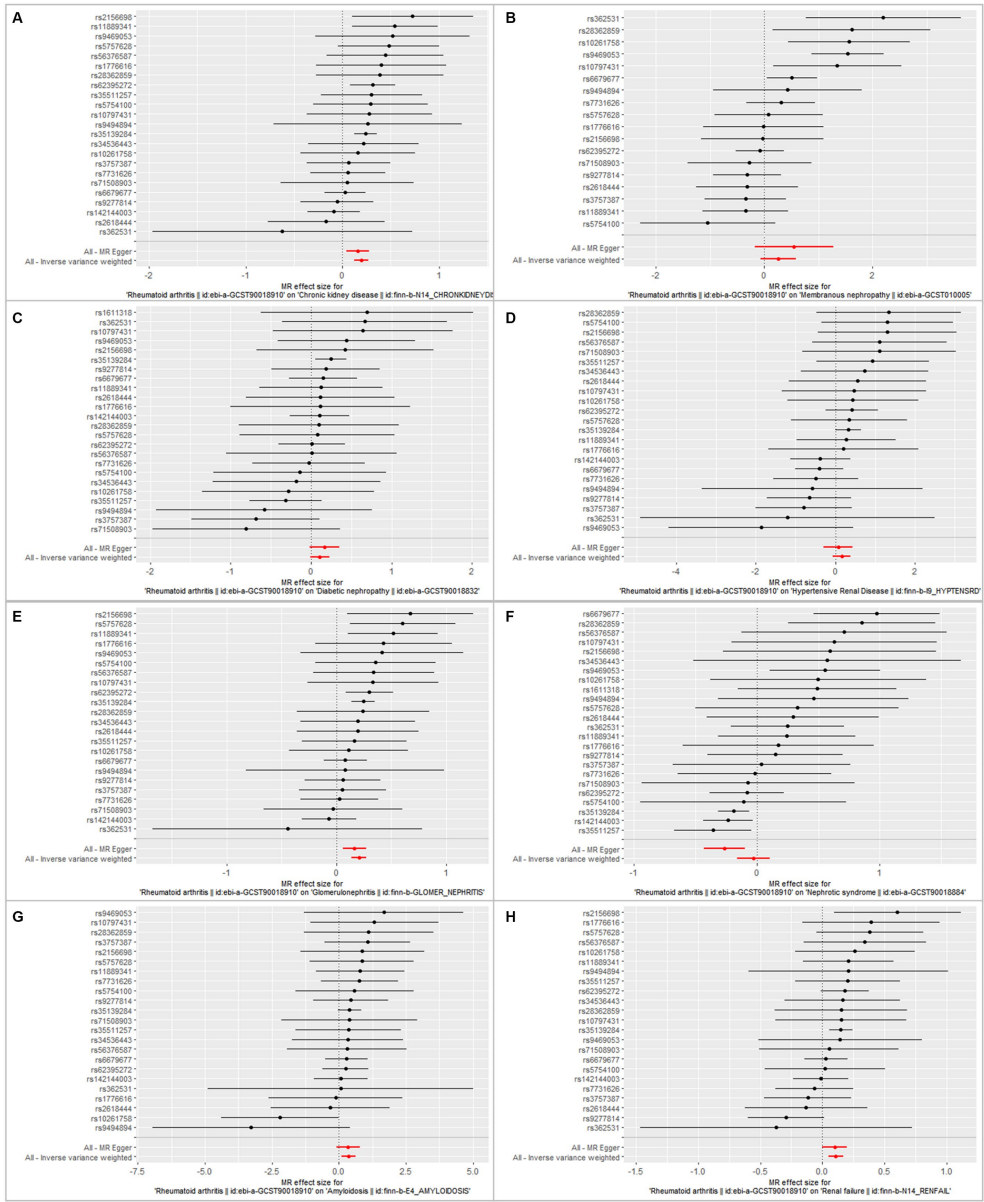
Forest plot showing the association between RA and CKD/CKD subtypes using instrumental variables of each SNP, alone or with all of the SNPs, using the MR–Egger or inverse-variance weighted methods. Bars show effect size and 95% confidence interval. **(A)** Chronic kidney disease. **(B)** Membranous nephropathy. **(C)** Diabetic nephropathy. **(D)** Hypertensive renal disease. **(E)** Glomerulonephritis. **(F)** Nephrotic syndrome. **(G)** Amyloidosis. **(H)** Renal failure.

In this MR analysis study, five methods were applied to confirm the robustness of the results, including the IVW method (the main method), MR Egger, weighted median, simple mode, and weighted mode (Figure 2). Based on the data from these methods, it can be inferred that a positive association existed between RA and CKD. By using the IVW method, a positive association was seen between RA and CKD (OR = 1.22, 95% CI 1.13–1.31, *p* < 0.001; Figure 2). The positive relevance was uniform when determined using the MR–Egger method (OR = 1.18, 95% CI 1.05–1.32, *p* = 0.013), weighted median (OR = 1.26, 95% CI 1.14–1.41, *p* < 0.001), simple mode (OR = 1.33, 95% CI 1.06–1.68, *p* = 0.023), and weighted mode (OR = 1.26, 95% CI 1.30–1.40, *p* < 0.001; Figures 2, 3). Furthermore, the results of sensitivity analyses, including forest plot and leave-one-out analysis showed that no SNP had a significant effect (Figure 4). In addition, MR–Egger did not detect potential horizontal pleiotropy for RA (*p* = 0.492), and neither the IVW nor MR-Egger methods revealed significant heterogeneity by Cochran’s Q test (*p* = 0.449, *p* = 0.418, respectively; Figures 2–6; Table 2).

**Table 2:** The sensitivity analysis results for the association between RA and relevant kidney diseases.

Exposure	Outcome	Method	Q	Q_df	Q_pval
RA	Chronic kidney disease	MR-Egger	21.67800985	21	0.418
		Inverse-variance weighted	22.18381	22	0.449
RA	Glomerulonephritis	MR-Egger	19.35860869	21	0.562
		Inverse-variance weighted	20.29785635	22	0.564
RA	Hypertensive Renal Disease	MR-Egger	25.76198573	21	0.216
		Inverse-variance weighted	26.21100151	22	0.243
RA	Diabetic nephropathy	MR-Egger	17.1068927	22	0.757
		Inverse-variance weighted	17.83548228	23	0.766
RA	Nephrotic syndrome	MR-Egger	39.50932544	22	0.012
		Inverse-variance weighted	62.85163718	23	1.46E-05
RA	Membranous nephropathy	MR-Egger	49.03590351	16	3.26E-05
		Inverse-variance weighted	51.50178119	17	2.46E-05
RA	Amyloidosis	MR-Egger	13.51462	21	0.8895324
		Inverse-variance weighted	13.52217	22	0.9175680
RA	Renal failure	MR-Egger	22.42732	21	0.3752545
		Inverse-variance weighted	22.47731	22	0.4317273

### The association between RA and CKD subtypes

By using the IVW method of MR analysis, a positive association existed between RA and glomerulonephritis (OR = 1.23, 95% CI 1.15–1.31, *p* < 0.000; Figure 2). Furthermore, the positive relevance was uniform when determined using the MR–Egger method (OR = 1.18, 95% CI 1.06–1.31, *p* = 0.006), weighted median (OR = 1.28, 95% CI 1.16–1.40, *p* < 0.001), and weighted mode (OR = 1.26, 95% CI 1.14–1.40, *p* < 0.001; Figures 2, 3). Potential horizontal pleiotropy was not detected for RA (*p* = 0.344), and there was no significant heterogeneity detected using Cochran’s Q test with either the IVW or MR–Egger methods (*p* = 0.564, *p* = 0.562, respectively; Table 2; Figure 4).

Furthermore, by using the IVW method of MR analysis, a positive association was seen between RA and amyloidosis (OR = 1.43, 95% CI 1.10–1.88, *p* < 0.001). Potential horizontal pleiotropy was not detected for RA (*p* = 0.932; Figures 2, 3), and there was no significant heterogeneity detected using Cochran’s Q test with either the IVW or MR–Egger methods (*p* = 0.918, *p* = 0.890, respectively; Table 2; Figure 4). Furthermore, a positive association existed between RA and renal failure (OR = 1.18, 95% CI 1.00–1.38, *p* < 0.001), the potential horizontal pleiotropy was not detected for RA (*p* = 0.831; Figures 2, 3), and there was no significant heterogeneity detected using Cochran’s Q test with either the IVW or MR–Egger methods (*p* = 0.432, *p* = 0.375, respectively; Table 2; Figures 4–6).

However, the other CKD subtypes were not significantly associated with RA, membranous nephropathy (OR = 1.29, 95% CI 0.93–1.79, *p* > 0.05), diabetic nephropathy (OR = 1.11, 95% CI 0.99–1.25, *p* > 0.05), hypertensive renal disease (OR = 1.17, 95% CI 0.94–1.47, *p* > 0.05), and nephrotic syndrome (OR = 0.97, 95% CI 0.85–1.11, *p* > 0.05; Figures 2–6; Table 2).

### The description of SNPs selected from these diseases

Comprehensively, the chronic kidney diseases significantly associated with RA, as analyzed using the MR analysis in this study included chronic kidney disease, glomerulonephritis, amyloidosis, and renal failure. The SNPs associated with these four diseases in the dataset were tested for single-nucleotide results, and a *p*-value of <0.05 indicated significant differences. The results are shown in Table 3, laying the foundation for subsequent correlation studies and analyses.

**Table 3:** Description of SNPs selected from these diseases.

Exposure	Outcome	SNP	b	se	p
RA	Chronic kidney disease	rs11889341	0.543831169	0.227272727	0.016717749
		rs2156698	0.727516779	0.318120805	0.022200503
		rs35139284	0.240950436	0.058659736	0.00004
		rs62395272	0.312778131	0.119834711	0.009052116
RA	Glomerulonephritis	rs11889341	0.517045455	0.209415584	0.013549415
		rs2156698	0.67114094	0.29261745	0.021814751
		rs35139284	0.245405606	0.053833302	0.00000515
		rs5757628	0.604554865	0.245341615	0.013734436
		rs62395272	0.299109981	0.110298792	0.006691581
RA	Amyloidosis	rs10261758	−2.207407407	1.118518519	0.048437494
RA	Renal failure	rs2156698	0.602684564	0.259060403	0.019995896
		rs35139284	0.146463709	0.047707444	0.00214

## Discussion

RA is a chronic inflammatory autoimmune disorder that is characterized by joint and extra-articular manifestations (EAMs) and can cause systemic effects (23). The EAMs of RA were more common in patients with severe active disease, for example, the eyes, lungs, skin, heart, and kidney (24). The mortality of RA patients with EAM is 2.5-fold that of RA patients without EAM. In addition, previous studies have found that the prevalence of comorbidities in patients with arthritis is high, including CKD (25) and glomerulonephritis (26).

CKD is a chronic, immune-mediated, and systemic inflammatory disease, which is one of the common complications in RA patients (4, 27–30). Several conditions can be used to explain the relationship between CKD and RA, such as secondary amyloidosis, glomerulonephritis, or a drug-related cause (31, 32). However, the specific causes of CKD in a majority of RA patients are usually not identified. Hence, elucidating the risk factors for developing chronic kidney disease among them is critical. In addition, the associations of RA with various kidney diseases are attributed to chronic inflammation, drug exposure, and toxicity (32). Furthermore, only a few studies have described the prevalence of chronic kidney disease in patients with RA (3, 4, 33). Thus, there are limited data on CKD in RA (34). The association between chronic inflammation and the incidence of CKD in RA patients remains unclear (13). Thus, MR was used to explore the potential causal relationship between RA and CKD in this study.

MR analysis is a powerful instrument for examining the associations between exposures and outcome factors and helps to prioritize potential causal associations. In comparison to randomized controlled trials, MR analysis has the advantage of controlling confounding factors and overcoming the waste of human and material resources. In addition, compared to observational studies, MR studies are generally considered to provide higher-quality evidence (16). The main virtue is the MR analysis study design, which confirmed the causal inference in the relationship between RA and CKD. This study used data from the IEU Open GWAS database with large sample sizes. In addition, this study included seven other CKD subtypes to confirm the relationship of RA. Finally, the results of the sensitivity analyses ensured the robustness of the associations. The SNPs with statistical significance shown in Table 3, established a foundation for further research and exploration.

Nonetheless, there were certain limitations in this study. The sample size is a common and important limiting factor in such studies, and there is more reliability in the conclusions when the sample size is larger. Furthermore, the data included mixed gender loci and since it is difficult to isolate gender data, gender may cause a bias in the current study, and may have influenced the results to some extent. For further study, a long-term cohort study is needed to validate the conclusions of this study, involving age and gender data. Additionally, the study samples were from individuals of European descent, so the generalizability of the MR analysis results to other races/ethnicities is uncertain. In addition, although confounders were removed through the PhenoScanner V2, the elaboration of specific drug effects was not clear, and therefore, the effects of drugs will be a key direction for subsequent exploration and research.

## Conclusion

In conclusion, the findings revealed that there is a causal association between RA and CKD, and in this MR analysis, glomerular nephritis, amyloidosis, and renal failure were found to be related to RA. Therefore, in RA patients, it is necessary to pay attention to the development of these types of chronic kidney diseases. However, it is unclear whether this causality is direct or indirect, and drug effects have not been completely ruled out, hence, further research is required. It is necessary to conduct large-scale prospective cohort studies to validate the preliminary findings of our study, and even more basic experimental studies with human tissues are needed to elucidate the deep molecular mechanisms.

To conclude, long-term follow-up of kidney function should be emphasized in RA patients to have the opportunity for earlier interventions to slow down the progression of CKD.

## Data availability statement

The original contributions presented in the study are included in the article/Supplementary material, further inquiries can be directed to the corresponding author.

## Author contributions

ZJ: Conceptualization, Methodology, Visualization, Writing – original draft. LC: Data curation, Investigation, Methodology, Writing – original draft. AL: Data curation, Methodology, Writing – original draft. JQ: Data curation, Methodology, Writing – original draft. JW: Investigation, Validation, Writing – original draft. YL: Data curation, Writing – original draft. HJ: Methodology, Writing – original draft. JZ: Formal analysis, Writing – original draft. SH: Project administration, Writing – original draft. CM: Project administration, Writing – original draft. ZY: Supervision, Writing – review & editing, Project administration.

## Footnotes

^1^ https://gwas.mrcieu.ac.uk

^2^ https://www.ebi.ac.uk/gwas/
